# Vehicular Visible Light Positioning System Based on a PSD Detector

**DOI:** 10.3390/s24072320

**Published:** 2024-04-05

**Authors:** Fatima Zahra Raissouni, Álvaro De-La-Llana-Calvo, José Luis Lázaro-Galilea, Alfredo Gardel-Vicente, Abdeljabbar Cherkaoui, Ignacio Bravo-Muñoz

**Affiliations:** 1 Department of Electronics, University of Alcalá, Alcalá de Henares, 28801 Madrid, Spain; alvaro.llana@uah.es (Á.D.-L.-L.-C.); alfredo.gardel@uah.es (A.G.-V.); ignacio.bravo@uah.es (I.B.-M.); 2 Laboratory of Innovative Technologies, National School of Applied Sciences, Abdelmalek Essaâdi University, Tangier 90060, Morocco; abcherkaoui@uae.ac.ma

**Keywords:** visible light communication, visible light positioning, vehicle-to-vehicle, angle of arrival, positioning sensor device, smart cities

## Abstract

In this paper, we explore the use of visible light positioning (VLP) technology in vehicles in intelligent transportation systems (ITS), highlighting its potential for maintaining effective line of sight (LOS) and providing high-accuracy positioning between vehicles. The proposed system (V2V-VLP) is based on a position-sensitive detector (PSD) and exploiting car taillights to determine the position and inter-vehicular distance by angle of arrival (AoA) measurements. The integration of the PSD sensor in vehicles promises exceptional positioning accuracy, opening new prospects for navigation and driving safety. The results revealed that the proposed system enables precise measurement of position and distance between vehicles, including lateral distance. We evaluated the impact of different focal lengths on the system performance, achieving cm-level accuracy for distances up to 35 m, with an optimum focal length of 25 mm, and under low signal-to-noise conditions, which meets the standards required for safe and reliable V2V applications. Several experimental tests were carried out to validate the results of the simulations.

## 1. Introduction

Improving driving safety is a critical objective in the development of intelligent transportation systems, as highlighted by the initiatives of various industry and government agencies [1]. The enhancement of ITS depends on two essential elements: efficient V2V communication and accurate vehicle positioning. These components are vital for ensuring the safety, efficiency, and reliability of vehicular interactions in an environment that is increasingly connected and automated.

Visible light communications (VLC) have emerged as a significant technology for overcoming these challenges by employing visible light for both data transmission and accurate positioning [2]. Offering advantages over conventional technologies like GPS, radar, and lidar, VLC stands out for its data security, cost-effectiveness, and practicality [3]. Its application is particularly valuable in outdoor settings for ITS, where it enhances both positioning accuracy and V2V communication reliability. Unlike traditional communication methods, VLC operates on an underutilized spectrum, reducing interference and network congestion [4]. Its directional nature also enhances data security by limiting signal dispersion, making it more difficult for signals to be intercepted. In addition, VLC can be integrated into existing vehicle lighting infrastructures, such as headlights and taillights, making it an economical and practical solution. As well as reducing hardware complexity, it also takes advantage of the energy-efficient LED lighting already available in many modern vehicles.

In the V2V, ITS, and smart cities (SC) context, measuring the distance and orientation between vehicles is crucial [5]. It enables the assessment of the relative position of vehicles and the anticipation of potential movements, thus reducing the risk of collisions. This measurement is particularly relevant for V2V-VLC, where the accuracy of relative position is essential to ensure reliable data transmission. V2V-VLC offers unique advantages over other wireless communication methods. It is less susceptible to RF interference and can provide a more secure communication channel [6]. However, VLC requires a clear line of sight between transmitters and receivers, making precise measurement of distance and orientation even more important. This precision optimizes connection quality and ensures efficient communication between vehicles.

Among the sensors commonly mentioned in the literature on V2V-based VLC technology, we find cameras [7] and photodiodes [8,9]. Although they have been frequently used in previous research for their ability to process light signals, they present certain limitations in the specific context of V2V-VLC.

While cameras are useful for image capture and pattern recognition, they suffer from relatively slow processing speed and sensitivity to light variations [4], which can compromise reliability in V2V scenarios where rapid decisions are crucial. Photodiodes, on the other hand, while effective for fast data transmission thanks to their high sensitivity, are limited by their small detection area and directionality, posing problems in dynamic V2V environments.

In our research, we have opted for a PSD sensor due to its exceptional ability to accurately detect the position and intensity of light signals [10], crucial for directional information and vehicle localization in V2V systems based on VLC. The PSD is a type of photodiode with a large surface area consisting of a single cathode and four anodes. This characteristic allows the determination of the impact point on its surface through the value of the currents provided for each anode. The use of discrimination techniques enables the differentiation between signals from multiple emitters. Despite its large surface area, compared to single-anode photodiodes, the sensor noise does not make a substantial difference. What changes is the capacitance between terminals, which has a greater impact on the frequency response bandwidth (BW). However, a PSD facilitates the use of larger lenses to collect energy on the sensor and capture more signal power.

The PSD’s high sensitivity and quick response align with the critical demands of V2V-VLC communication for enhanced accuracy and low latency, addressing the challenges of maintaining line of sight and precise alignment in the case of obstructions or potential deviations. This capability enhances adaptability to dynamic road situations, such as lane shifts or speed changes, improving the communication range and reliability by ensuring optimal alignment. Our exploration focuses on leveraging VLC within ITS, particularly in V2V information exchanges, by incorporating PSDs to advance vehicle communication and localization, contributing significantly to future transportation systems’ safety, efficiency, and dependability.

It should be noted that in this work, we focus on the fundamental aspects of VLP localization by following the framework established by standards such as IEEE 802.15.7 [11]. Although frequency discrimination is a relevant topic, our study aligns with these existing protocols which do not explicitly address this issue. This approach allows us to focus our resources on validating the key concepts of VLP localization, while recognizing that frequency discrimination represents an interesting avenue for future research. It should be noted that if the methods for discriminating sequences and frequencies are well chosen, there is no significant interference between the different agents, as has been reported in previous work by the group [12].

The rest of the paper is organized as follows: Section 2 presents the background and related work. Section 3 presents the mathematical model of the vehicular VLP system and the setup of AOA measurements. Section 5 presents the results and discussion. In conclusion, a summary of the contributions can be found in Section 6.

## 2. Background

Several studies have explored a range of different techniques in the field of vehicular visible light positioning (VVLP), contributing to the current state-of-the-art methods for the development of VVLP. The TDoA (time difference of arrival) method [13] uses VLC signals from traffic lights to estimate the position of vehicles, providing efficient self-location, but with a limited accuracy of about 1 m and dependent on the presence of localized traffic lights. The PDoA (phase difference of arrival) method [8,14] achieves centimeter-level accuracy using the phase difference of the VLC signals of the front/rear lights, but it is based on the assumption of parallel orientation of the vehicles and requires high frequencies that are not compatible with standard automotive LEDs.

Similarly, RToF (range time of flight) [15,16] is based on the time of flight of a VLC message between two vehicles for precise longitude positioning. However, this method suffers from poor lateral accuracy and requires special broadband circuits. Faced with these challenges, the AoA approach [17,18] stands out for its promise of high accuracy without imposing restrictions such as limited vehicle orientations or the presence of traffic lights. However, current AoA implementations are mainly limited to camera-VLC-based methods, which are either slow in terms of communication rates or expensive due to the requirement for high-frequency cameras. The authors in [19] introduced a VLP technique using a single quadrant photodiode (QPD) to determine the position of neighboring vehicles through AOA measurements. However, the proposed method requires the target vehicle to actively broadcast its heading and speed information via VLC. This requirement could limit the applicability of the method in real-world environments, where not all vehicles are equipped with or constantly transmit such information. Furthermore, some studies, e.g., [20,21], have explored the use of LED street lights to transmit identification codes accompanied by real coordinates. These codes are captured by CMOS image sensors installed in the vehicles, making it easier to determine their position. Nevertheless, it should be noted that the image processing algorithms required for this purpose are characterized by significant computational complexity [20].

The research group to which the authors of the article belong has extensive experience in investigating positioning systems based on infrared and visible light. This expertise extends to the design and enhancement of PSD sensors. Traditionally, PSDs have been used for high-precision positioning of offsets, alignment variations and angle variations between two systems where the relative positioning variation is very small. In these cases, PSDs are often used in combination with a laser with collimated beams and without coupling optical systems or lenses to the PSD detector, such as [22]. However, this type of application is different and not comparable to applications such as the one presented in this work. For some years now, our research group has been working on developing indoor positioning systems (IPS) in which the emitter, or the detector, moves freely (without being solidly or mechanically coupled to each other) in spaces of several metres in 2D or 3D. In these cases, the detector always has a lens coupled to it to form an image on the detector, which allows us to determine the angle of arrival of the light beam. In fact, it is possible to receive more than one beam of light and determine the angles of arrival of all of them with accuracy as long as they use different beam modulation frequencies [12].

This includes addressing the sources of electrical errors as outlined in [17], where strategies for correcting and mitigating these effects are discussed. Additionally, reference [23] provides a geometric model of the PSD sensor optical system and details a calibration process for extracting intrinsic parameters. As a result of these advancements, as reported in [10], the team has developed a high-precision indoor positioning system (IPS) achieving accuracy to the order of millimeters. In addition, it should be noted here that the system developed based on PSD is not affected by ambient light, including sunlight, since the continuous component and the low frequencies of the incident signals are filtered. On the other hand, the fact of using a PSD and not a photodiode makes the effects of multipath practically negligible, since AoA measurement techniques can be applied. Photodiodes, however, when performing signal power and phase measurements only, cannot compensate for these effects and are greatly affected, as has been studied in previous work of the group.

In the present work, we have designed a V2V positioning system for determining position and estimating distance and lateral deviation using PSD sensors and angle of arrival (AoA) measurements.

To the best of our knowledge, this is the first time that a PSD sensor has been used in a V2V-VLC application. Our choice of the PSD sensor is based on its potential to significantly improve the quality and performance of V2V-VLC communication systems, paving the way for future developments in this field.

## 3. System Model and Vehicle Localization

### 3.1. System Model

**Figure 1:**
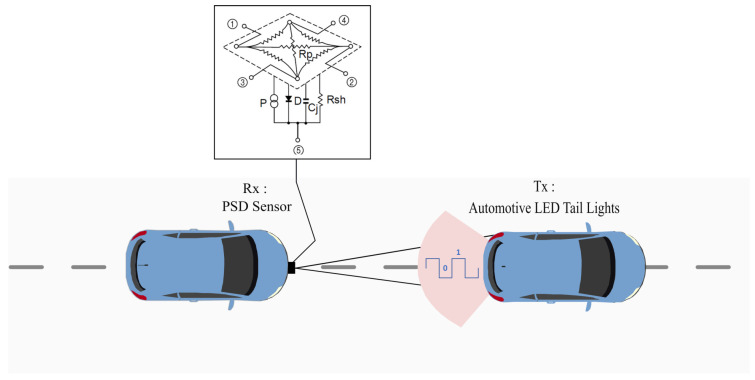
Scheme of positioning system and pincushion PSD model. Numbers 1, 2, 3 and 4 in the PSD equivalent circuit correspond to the anode terminals and number 5 to the cathode terminal.

This section presents the proposed system model, including both transmitter and receiver. In this setup, VLC transmitters use the existing light sources on vehicles, such as headlights and taillights, which are adapted to emit VLC signals. At the same time, a PSD receiver is installed on the following vehicle to capture the light signals emitted by other vehicles. The function of the PSD extends to detecting the location of the incoming light beam on its surface, enabling the precise direction of arrival of the signal to be determined. Figure 1 also shows a model of a two-dimensional pin-cushion PSD sensor.

The PSD sensor has four anode pins and a common cathode pin. The theoretical point of impact of a light beam from the emitter, collected through a lens, is determined from the output of each anode according to Equations (1) and (2) as shown in [23]:

(1) $$\begin{array}{c} x=f\left(L_{X},I_{X1},I_{X1},I_{Y1},I_{Y2}\right), \end{array}$$

(2) $$\begin{array}{c} y=f\left(L_{Y},I_{X1},I_{X1},I_{Y1},I_{Y2}\right), \end{array}$$

where $I_{X1},I_{X2},I_{Y1},$ and $I_{Y2}$ are the electrical currents from the PSD sensor anode pins and $L_{x}$ y $L_{y}$ are the sensor dimensions. To characterize the sensor + optical unit, the pinhole model was used, which allows us to obtain the geometric parameters of the system and to know the amount of energy that is collected [23].

The PSD presents a fast-processing alternative that offers a series of features over conventional cameras and photodiode arrays, including fast response time, good positioning accuracy and simple signal conditioning circuitry [24]. It should be noted that the advantage of PSD-based systems lies in their ability to provide precise information on the position of the light source, which is an essential parameter in V2V communication for safety and navigation purposes. In contrast, VLC MIMO systems focus on improving data throughput and reliability [25].

**Figure 2:**
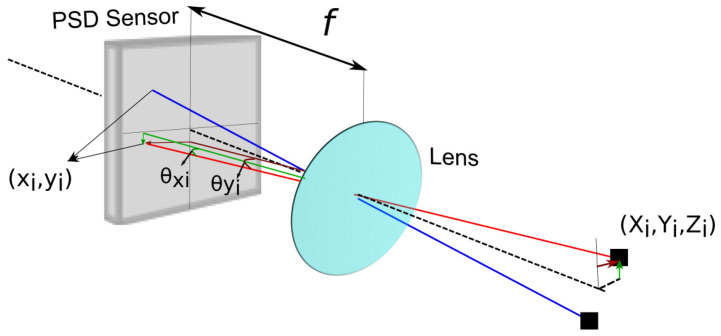
PSD sensor with lens. Black line represents the optical axis of the system. Red and blue lines represent the translation of points in 3-D space to their image on PSD detector.

A conceptual diagram of the PSD and its optical configuration are shown in Figure 2. The PSD is specifically designed for high-resolution AoA measurement rather than simply achieving angular diversity. The PSD has an added lens placed at a certain distance (focal distance) above a PSD converging the rays from the TX LED into a defocused spot.

In Figure 2, $\left(x_{i},y_{i}\right)$ represent the impact points on the PSD sensor, *f* is the focal length, $\left(X_{i},Y_{i},Z_{i}\right)$ are the points in the transmitters in the environment, $\left(\theta_{\mathrm{xi}},\theta_{\mathrm{yi}}\right)$ are the angles of arrival (components of AoA), and *i* represents each emitter, using Equations (3) and (4) to calculate the angles:

(3) $$\begin{array}{c} \theta_{\mathrm{xi}}=\arctan\left(\frac{x_{i}}{f}\right), \end{array}$$

(4) $$\begin{array}{c} \theta_{\mathrm{yi}}=\arctan\left(\frac{y_{i}}{f}\right), \end{array}$$

As can be seen, the field of vision is related to the size of the PSD sensor and the focal length of the lens, according to Equation (5):

(5) $$\mathrm{FoV}=2\arctan\left(\frac{L}{2f}\right),$$

where *L* is the diagonal of the sensor and *f* is the focal length of the lens. The system model, which correlates the 3D positions of the emitters in the world with their corresponding impact points on the sensor’s surface $\left(x_{i},y_{i}\right)$, is detailed in reference [23]. The points of the coordinates in the real-world coordinate system are $\left(X_{w},Y_{w},Z_{w}\right)$, and $\left(X_{R},Y_{R},Z_{R}\right)$ are the coordinates of the emitters referring to the PSD system.

(6) $$\left(\begin{array}{c} X_{R}\\ Y_{R}\\ Z_{R} \end{array}\right)=R\left(\begin{array}{c} X_{W}\\ Y_{W}\\ Z_{W} \end{array}\right)+T,$$

The relationship between the receiver’s reference system and the image plane is represented by Equation (7)

(7) $$\left(\begin{array}{c} sx\\ sy\\ s \end{array}\right)=\left(\begin{array}{ccc} f & 0 & C_{x}\\ 0 & f & C_{y}\\ 0 & 0 & 1 \end{array}\right)\left(\begin{array}{c} X_{R}\\ Y_{R}\\ Z_{R} \end{array}\right),$$

where *s* represents the scale factor that relates the 3D to 2D projection. The matrix system in Equation (7) presents the mathematical model of the receiver, without considering the distortions produced by the lens and the PSD sensor. By combining Equations (6) and (7) we obtain:

(8) $$\left(\begin{array}{c} sx\\ sy\\ s \end{array}\right)=\underset{A}{\underset{︸}{\left(\begin{array}{ccc} f & 0 & C_{x}\\ 0 & f & C_{y}\\ 0 & 0 & 1 \end{array}\right)}}\underset{\mathrm{RT}}{\underset{︸}{\left(\begin{array}{cccc} r_{11} & r_{12} & r_{13} & T_{x}\\ r_{21} & r_{22} & r_{23} & T_{y}\\ r_{31} & r_{32} & r_{33} & T_{z} \end{array}\right)}}\left(\begin{array}{c} X_{W}\\ Y_{W}\\ Z_{W}\\ 1 \end{array}\right),$$

(9) $$\left(\begin{array}{c} sx\\ sy\\ s \end{array}\right)=\mathrm{ART}\left(\begin{array}{c} X_{W}\\ Y_{W}\\ Z_{W} \end{array}\right),$$

where *A* presents the matrix values of the intrinsic parameters and RT is the rotation-translation matrix. Furthermore, the authors in [23] introduce a calibration technique to determine the intrinsic parameters of the measurement system.

#### Problems That Arise Developing the System with PSD

In order to obtain a good measurement and ensure accuracy of the AoA using PSD, several issues need to be taken into account.

Given that the emitters are located at large distances from the PSD detector and that their emission pattern is divergent, the energy that reaches the detector is very small and, therefore, so is the current it generates (of the order of nA-uA); therefore, it must be amplified to be able to work with them. Furthermore, all the error sources that each PSD channel may have must be calibrated together with the error sources that the external circuits may present. The amplification circuits may present imbalances in their gains, so a calibration process will be needed to adjust all possible sources of error and make each of the four channels of the PSD, including its amplification circuits, behave exactly the same since the signals delivered will be used to obtain the impact point of the light beam image and, therefore, the AoA [26].

In the case of the signal conditioning circuits, we use transimpedance amplifiers as the first stage to amplify the signals delivered by the four anodes and perform the current–voltage conversion.

As can be seen, the circuit allows working with output voltages Equations (1) and (2) instead of currents.

In addition, a band-pass filter (BPF) is added to eliminate noise at frequencies higher than the modulation frequencies of the emitters.

Additionally, several sources of error are present when calculating the point of incidence on the surface of the PSD sensor [26] and they must be analyzed:

- Component tolerances (feedback resistor and amplifier stage capacitor) affect the variance of the gains. Each of the four output PSD signals is, therefore, amplified by a $k_{i}$ factor. This amplification will cause a deviation in the determination of the actual point of impact. Both tolerances should be modeled on a uniform distribution U[a,b] using realistic values for components and their tolerances. In addition, $k_{i}$ will depend on the frequency. Although the frequency used for this PSD is less than 50 kHz, the frequency and tolerances will be analyzed together. These factors will provide a relevant error in the total error analyzed, even using low tolerance values.
- Temperature changes in the gain component values. Different profiles of operating temperatures and temperature component coefficients based on realistic values should be used in the simulation and test phases. A uniform distribution U[a,b] should be modeled to incorporate the above characteristics. Changes in temperature will cause changes in the nominal values of the components. However, it can be considered as an independent effect of the tolerance components. For this situation, it is estimated that the generated error will not be a notorious contribution because the temperature effect will have the same influence in all system components.
- Influence of system noise. There will be a correlation between them. The different types of noise to be evaluated will be: shot, thermal, and operational amplifier noise. All will be analyzed under a white Gaussian noise hypothesis $\left(N\left(0,\sigma^{2}\right)\right)$.
- The shot noise depends on the dark current and the sensor (photodiode) current. This noise source will be the main contribution to the total error of this stage. Although the PSD sensor used in this work (pin-cushion) produces less dark current than others, the shot noise will mainly be the most notable error.
- Thermal noise depends on the component values, temperature variations, and equivalent bandwidth. This type of noise is correlated with the previous analysis (component tolerance and temperature variations). The degree of correlation between them depends on the tolerance and temperature variations, but the contribution of the shot noise (independent of them) has more influence than this. In order to simplify the analysis, each should be evaluated separately. The influence of this error in the impact sensor point depends on the SNR.
- Amplifier noise does not depend on external parameters. It will only depend on the type of operational amplifier. For this reason, we suggest choosing a low noise FET type. The use of band-pass filters to reduce the signal noise eliminates the offset, so bias currents and offset voltages and currents at the amplifier input do not affect the system.

Other sources of noise to be analyzed are those from the analog-to-digital converter. Quantization errors come from the number of bits in the analog-to-digital converter. This type can be reduced by increasing the number of bits, optimizing the SPAN or increasing the sampling frequency.

Here, we propose a method of measuring the angle of arrival using a PSD sensor to determine the distance between vehicles. To this end, given that an optical system is attached to the PSD with non-ideal behaviour with respect to the theoretical pinhole model used, it was necessary to perform a geometric calibration to determine the intrinsic parameters of the system, thus allowing us to accurately determine the angle of arrival of the beam [23].

This approach is based on obtaining the relationship between the environment and the optical system. Since it does not adjust the intrinsic parameter values (optical center, focal length, and distortion parameters), the distance measurement will contain an error that can only be corrected by calibrating the real intrinsic parameters. The needed PSD sensor system geometric calibration using mathematical models is based on the pin-hole model. This is a linear model that relates points in the environment to points in the image, depending on the intrinsic linear parameters of the optical system (without distortion, since the model does not have a lens), and the extrinsic linear parameters (rotation, translation). However, despite the similarity, there are differences, because cameras are array detectors with millions of receiver cells (space is discretized), whereas a PSD is an analog sensor that delivers very small currents (pA), with all of the problems and errors that this entails. The parameters corresponding to the effects produced by the lens attached to the PSD sensor, such as radial and tangential distortion, were added to the pin-hole model, as were the corresponding distortion parameters presented by the PSD sensor itself [23].

### 3.2. Vehicle Localization with AOA-Based VLP

**Figure 3:**
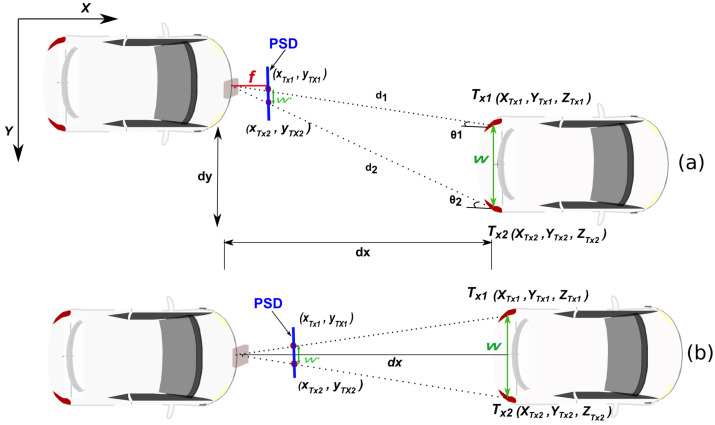
Triangulation method to obtain the distance between vehicles: (**a**) Scenario A; (**b**) Scenario B.

This section presents the AoA-based VLP system that is used for vehicle localization and positioning accuracy which represents the sensitivity of the dual-AoA measurements. AOA is a triangulation method that relies on measuring angles from transmitters placed at specific locations in order to deduce the position of a moving target device. Figure 3 shows the triangulation method to obtain the inter-vehicular distance dx. In image systems mounting lenses, it is common practice to redefine the schema and place the sensor in front of the lens to simplify the view and avoid the need to display the image inverted. Following that schema, the PSD has been depicted in front of the lens in Figure 3 instead of behind the lens as it is really placed, as shown in Figure 2 (where the image is inverted).

The distance between the two rear lights is denoted by W, while its projection on the PSD surface is denoted by w′. The parameter *f* represents the focal length of the sensor (very enlarged in the figure to show it conveniently and to be able to see it, since it is only a few mm in length).

- Scenario A: the leading vehicle with two rear light emitters and the following vehicle with a PSD sensor. The vehicles are not perfectly aligned, introducing a lateral offset. The distance between the two rear lights (emitters) is known. Let us define dx as the longitudinal distance from the PSD to the midpoint between the two rear lights. *w* represents the distance between the two rear lights. dy presents the lateral offset—the distance by which the following car is displaced to the side relative to the leading car’s centerline.

- Scenario B: (Vehicles aligned, dy=0). In this case, the cars are aligned on the horizontal axis and there is no lateral component (Y=0). We can estimate the inter-vehicular distance dx using the known width between two taillights and the projection of these points on the PSD sensor.

To compute the inter-vehicular distance, $d_{x}$, the following expression can be used, as depicted in Figure 3.

(10) $$\begin{array}{c} d_{x}=f\frac{W}{w\prime }=f\frac{\sqrt{{\left(X_{\mathrm{TX}1}-X_{\mathrm{TX}2}\right)}^{2}+{\left(Y_{\mathrm{TX}1}-Y_{\mathrm{TX}2}\right)}^{2}}}{\sqrt{{\left(x_{\mathrm{TX}1}-x_{\mathrm{TX}2}\right)}^{2}+{\left(y_{\mathrm{TX}1}-y_{\mathrm{TX}2}\right)}^{2}}}, \end{array}$$

(11) $$\begin{array}{c} d_{1}=\frac{d_{x}}{\cos(\theta_{1})}, \end{array}$$

(12) $$\begin{array}{c} d_{2}=\frac{d_{x}}{\cos(\theta_{2})}, \end{array}$$

where $X_{\mathrm{TX}1}$ and $Y_{\mathrm{TX}1}$ are obtained from Equation (9) by substituting $X_{W}$, $Y_{W}$, and $Z_{W}$ with the coordinates of emitter 1. Similarly, $X_{\mathrm{TX}2}$ and $Y_{\mathrm{TX}2}$ are obtained from the coordinates of emitter 2. Equation (10) and its explanations are theoretical. However, in our specific scenario, what we obtain are the points of impact within the PSD surface of the two emitters $\left(X_{\mathrm{TX}1},\;Y_{\mathrm{TX}1},\;X_{\mathrm{TX}2},\;Y_{\mathrm{TX}2}\right)$. From this information, we derive $d_{x}$ and $d_{y}$.

The lateral distance, $d_{y}$, can be estimated using the vertical position of the image of the reference points on the sensor and considering the geometry of the situation. If $y_{p}$ is the vertical position of the image of the reference points on the camera sensor, then $d_{y}$ can be calculated as follows:

(13) $$\begin{array}{c} d_{y}=y_{p}\frac{d_{x}}{f}, \end{array}$$

## 4. Methodology for Performing Simulations and Measurements

**Figure 4:**
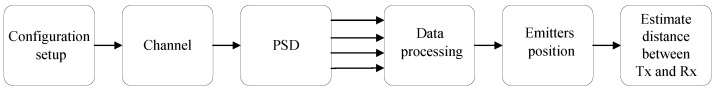
Flowchart that summarizes the steps of the V2V-VLP simulation process.

Figure 4 shows a flowchart summarizing the process used to calculate the receiver’s position from the signals detected by the PSD.

**Figure 5:**
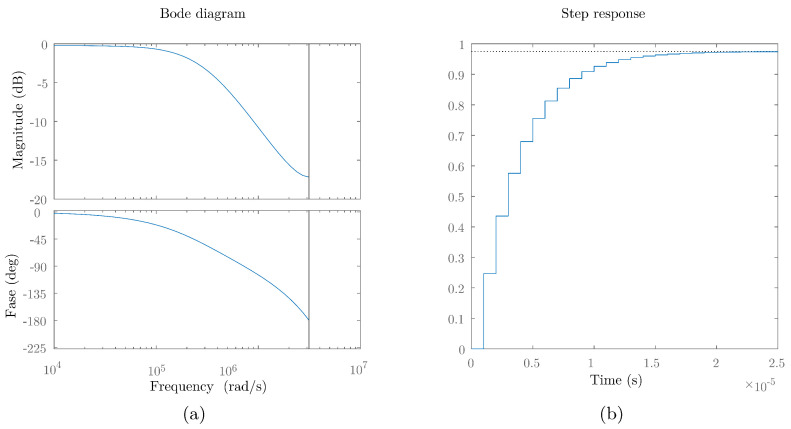
Transfer function of PSD sensor. (**a**) Bode, (**b**) Step response.

- Configuration Setup. The configuration setup concerns how the general parameters of the system are configured, such as the sampling frequency, modulation type, number of transmitters, and the characteristics of both the transmitter and the receiver. This essential configuration paves the way for the next phase, the generation of the emitted signal, where the signal that will be emitted by each of the emitters is generated depending on the type of modulation and technique defined previously.
- Channel: In this stage, the behavior of the channel is simulated from the moment the signal is emitted by the emitters until it is finally received by the PSD sensor.
- PSD:
  1. In this step, the impact point of each of the emitters on the surface of the PSD sensor is obtained from the coordinates and orientations of both the emitters and the PSD receiver.
  2. Attenuation. At this stage, the signal that would reach the receiver is obtained, formed by the emitted signal attenuated according to the received power value.
  3. PSD transfer function. The signal coming from the previous stage is passed through the transfer function to obtain the real one that would be received by the PSD from each of the emitters. To model the behavior of the PSD, its transfer function was obtained experimentally. For this purpose, a step signal was emitted with a transmitter and this signal was received in the PSD. In Figure 5, the Bode diagram and the step response of the experimental PSD transfer function are shown.
    From the values in Figure 5, the experimental transfer function of the PSD was estimated. For the case of a sampling frequency of 1 MHz, the following transfer function was obtained:
    (14) $$\begin{array}{c} TF_{\mathrm{PSD}}\left(z\right)=\frac{0.2469z^{-1}}{1-0.7644z^{-1}+0.01766z^{-2}} \end{array}$$
    It is worth noting that in this simulation, a Hamamatsu S5991-01 PSD (Hamamatsu, Iwata City, Japan) with a surface area of 9 × 9 mm^2^ was modeled. This PSD obtains a bandwidth of 200 kHz, which is sufficient for a wide range of communication and/or positioning applications. However, it would be possible to use a smaller PSD (4 × 4 instead of 9 × 9) to increase the frequency response ×7 (up to 1.5 MHz) but the field of view would be half considering the same focal length in the lenses used.
  4. Amplitude of each channel as a function of the impact point. Depending on the impact point on the PSD surface, a different amplitude is generated at each of the sensor anodes. This amplitude is related to the distance between the impact point and the corner corresponding to the channel (Equations (1) and (2)).
  5. Signal from each transmitter and PSD channel. The signal after passing through the transfer function would correspond to the total signal (sum of the signals from the four channels) received by the receiver from each of the transmitters. To obtain the signal from each of the four channels from the total signal, the total signal is multiplied by a value $A_{c}$. This value $A_{c}$ is a value between 0 and 1, and their sum is 1 ($\sum_{c=1}^{4}A_{c}=1$). $A_{c}$ corresponds to the proportion of the total power that reaches each channel *c*, which depends on the impact point.
  6. Signal from each channel. At this stage, the contribution of each of the transmitters in each of the PSD channels is added and the continuous signal is eliminated.
  7. Noise generation. At this stage, all noise components will be combined into a single noise component. The noise considered is white Gaussian noise. Depending on the received signal and the SNR to be analyzed, a different noise signal will be generated. Therefore, when simulating the noise, the following strategy is followed: After capturing the signals received by each of the four channels of the PSD under conditions without noise, we proceed to compute the total power, denoted as Ps, of the received signals. Following this, we can determine the noise power $P_{n}$, represented as:
    (15) $$\begin{array}{c} Pn=\frac{P_{s}}{10^{SNR/10}} \end{array}$$
  8. For each channel of the PSD, we generate Gaussian noise with a zero mean and a noise power that is one-quarter of the total power. This noise generation is based on the following formula:
    (16) $$\begin{array}{c} \mathrm{noise}=\sqrt{\frac{P_{n}}{4}}\mathrm{randn} \end{array}$$
    employing randn, a MATLAB function that produces random numbers with a normal distribution. Subsequently, we add this generated noise to the signal of each of the four channels, effectively modifying each signal with its respective noise component.
- Calculate emitter position: The signal from each emitter is discriminated by each of the anodes of the PSD [12], and with that signal, the impact point of each emitter on the surface of the PSD is obtained. Once the impact point on the surface of the PSD is known, and based on the intrinsic and extrinsic parameters, the position of the emitters is estimated.
- Estimate distance between Tx and Rx: Estimating the longitudinal distance between cars (inter-vehicular distance) as well as their lateral separation.

It should be noted that for the propagation model, in all simulations, a lead target vehicle transmits VLC signals from its taillight to the following vehicle: this is the worst-case scenario, with *n* = 1 (Lambertian order) consideration, as taillights have lower optical power than headlights and also because a different *n* > 1 diagram concentrates more energy on the receiver. Furthermore, in line with ECE R112 regulations [26], our study considers the characteristics of low-beam lamps [27]. Moreover, in truck platooning and V2V positioning, where a short tracking distance is maintained, a low beam headlight becomes the suitable choice as a VLC transmitter. This choice is particularly beneficial in mitigating the effects of vertical oscillation, which is a common challenge in vehicular communication systems [28].

## 5. Results

In this section, we present the simulation results obtained using MATLAB© R2023a to evaluate the performance of the proposed V2V-VLP system in several different scenarios taking as a starting point Scenarios A and B already introduced in previous sections. The simulation parameters are listed in Table 1.

It is noted that the results presented in this section were obtained using a signal processing time of 0.04 s (corresponding to 40,000 samples at a rate of 1 MS/s). This value was chosen as it would provide a distance measurement rate of 25 measurements per second. It is important to highlight that the processing time used will affect the precision of the distance measurement. Adjusting this value, either reducing or extending it, will result in an increase or decrease in measurement precision, as a Gaussian noise with a mean of 0 is being used, which is commonly employed in such systems due to its faithful representation of reality. Since an exhaustive analysis of precision is beyond the scope of this work, the results of the variation in precision with processing time for an indoor positioning system with PSD can be found in [10]. Although the scenarios and applications may differ, those results are directly applicable to this study.

In addition to increasing the signal processing time, other techniques could be employed to minimize the distance measurement error. For instance, one approach could involve taking an average of the last n distance measurements for each measurement instance. That is, at time t, the distance measurement can be obtained as the average of the previous n values, and at time t + 1, a new value is obtained using the previous n values. This approach would maintain the same rate of distance measurements per second while reducing the error. However, the drawback of this approach is that the results would be “filtered” as they would depend on previous values. Depending on the application, this method could be valid, as well as adding other types of processing, such as a Kalman filter, for example. In this work, we have chosen to present the values without this type of post-processing as they represent a worst-case scenario, and any subsequent post-processing would improve the results.

### 5.1. Simulation Setup

**Figure 6:**
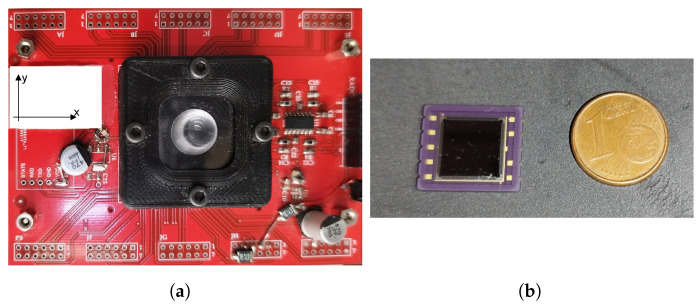
Proposed detector system (**a**) electronic board (**b**) Sensor module.

In our simulation setup, we have simulated the PSD sensor illustrated in Figure 6. The receiver used is a Hamamatsu S5991-01 PSD transducer (measured bandwidth: 200 kHz, nominal active area: 9 × 9 mm^2^). Figure 6a shows the PSD sensor mounted on an electronic board, designed to facilitate connection with the communication and signal processing systems. The x- and y-axes are shown to define the orientation of the sensor, crucial for detecting the direction of the VLC signal. Figure 6b shows the sensor next to a 1 euro cent coin to provide a reference scale, highlighting the sensor’s size.

The real size of the sensor is essential for our study, as it influences integration and deployment in realistic V2V environments. Its small size makes it easy to install in various locations on the vehicle, offering flexibility in experimental design.

In order to make the simulations as realistic as possible, as previously indicated, noise will be introduced into the signal. To properly quantify when to introduce noise, the noise of the boards we are going to emulate was measured. Specifically, the noise of two boards that we have available was measured: one with a one-inch diameter lens and an amplification gain of 20 million (board 1), and another board with a half-inch diameter lens and an amplification gain of 2 million (board 2). With the amplification gain of the boards known, the SNR that the received signal would have can be correctly adjusted based on the emitted power and the distance. This provides a quantitative measure of the signal quality in various test conditions. This analysis helps to identify the performance of the PSD sensor in different spatial configurations and under different noise levels.

From the measurements conducted, an RMS noise value of 40 mV was obtained for board 1 and 20 mV for board 2. With these values and the known amplification gain, emitted power, and distance, the SNR that would be obtained at each distance could be calculated. For example, using board 1 with an emission power of 15 W, a 1-inch lens, gain of 20 million, and at a distance of 25 m, an approximate SNR of 50 dB would be obtained, worsening to 30 dB at 70 m. Another example, and the one to be used in the experimental tests, using board 2 with an emission power of 5 W, a 1/2-inch lens, gain of 2 million, and at a distance of 25 m, an approximate SNR of 10 dB would be obtained. Since the SNR depends on the distance, emitted power, and characteristics of the receiver noise, various environments with SNR of 10, 20, and 50 dB will be simulated in this work.

In the following section, we present the simulation results. For each series of tests, we modify the critical parameters of the system, such as the focal length of the lens used and the SNR.

It is important to note that, for these simulations, we have adopted an SNR of 10 dB to represent the worst-case scenario. This approach is justified by our actual measurements, which indicate that there is a critical distance of more than 100 m using board 1 and 25 m using board 2, suggesting that our 10 dB simulations provide a conservative estimate of performance under the most challenging conditions. This precaution is particularly relevant given that, in platooning scenarios, vehicles typically maintain a distance of around 15 m, which would normally result in a higher SNR than our simulated worst-case scenario.

### 5.2. Simulation Results

**Figure 7:**
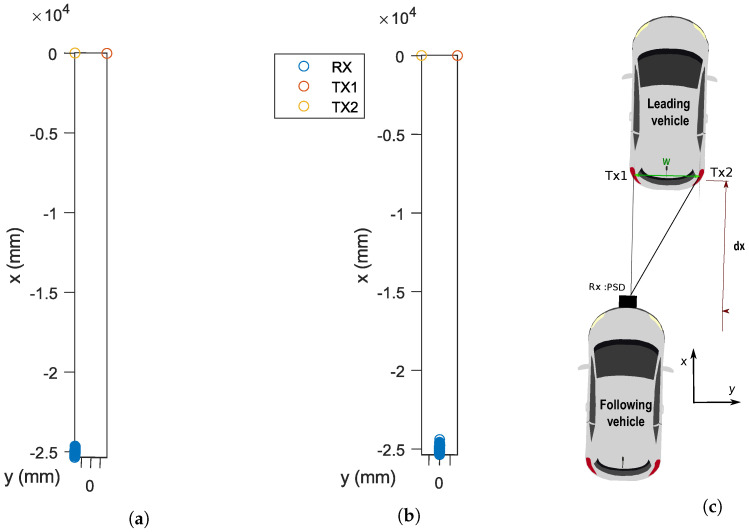
Position of the receiver and transmitters: (**a**) Scenario A; (**b**) Scenario B; (**c**) visual illustration of Scenario A.

To assess the accuracy of the proposed algorithm, we first need to simulate the positions of the transmitters and the receiver in the environment. Figure 7 illustrates the results of simulations designed to evaluate the localization accuracy of a vehicle positioning system for an SNR = 20 dB. The origin coordinates are anchored to the geometric center between the taillights, which is considered the essential reference point for localization. Given that the origin of the coordinates is in the center between the two taillights and the follower car is in this presentation at a distance of 25 m, for Scenario B, the two cars are aligned and there is a lateral deviation for Scenario A. This SNR was simulated to reproduce clear visibility conditions and minimal interference. The original position was set at the exact center between the taillights, which served as the reference point for all measurements. The measured x- and y-coordinates are presented in millimeters, with the y-axis indicating the lateral distance from the center and the x-axis the longitudinal distance.

To assess our proposal, simulations were conducted to understand its behavior under different conditions. Below, we present the results obtained from the evaluation, which include the dispersion in determining the receiver’s position, errors in longitudinal and lateral distance measurements at various distances, using different focal lengths, and various signal-to-noise ratios (SNRs). Since noise is a random variable, the experiments were repeated 100 times to analyze the results of all these parameters.

#### 5.2.1. Receiver Position Estimation

**Figure 8:**
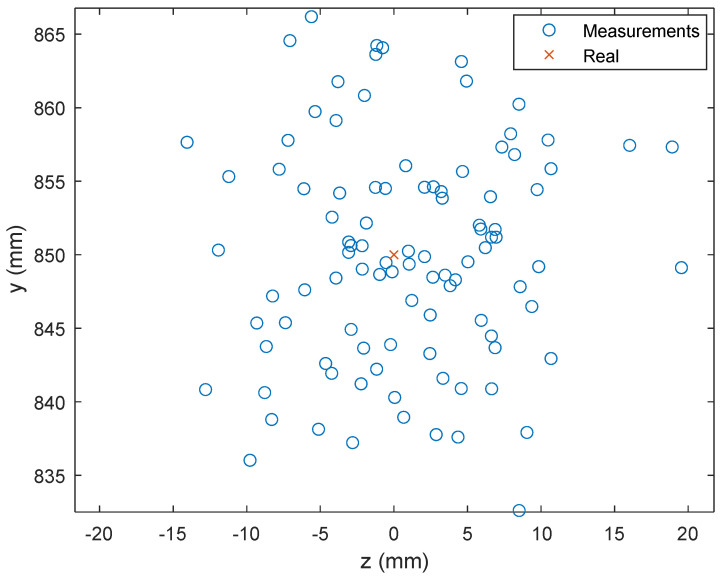
Estimated and true receiver positions with SNR = 20 dB and f = 25 mm and taillights separation W = 1.7 m.

Figure 8 shows the results of a series of 100 iterations of position measurements of a fixed receiver, located between the two headlights of a vehicle, in a visible light communication (VLC) system with an SNR of 20 dB. The distributed measurements (blue circles) around the actual position (red cross) reveal a substantial dispersion, indicating a significant influence of noise on the accuracy of the position estimates. The range of this dispersion extends up to 50 mm in both x- and y-directions.

#### 5.2.2. Inter-Vehicular Distance Estimation Scenario A

**Figure 9:**
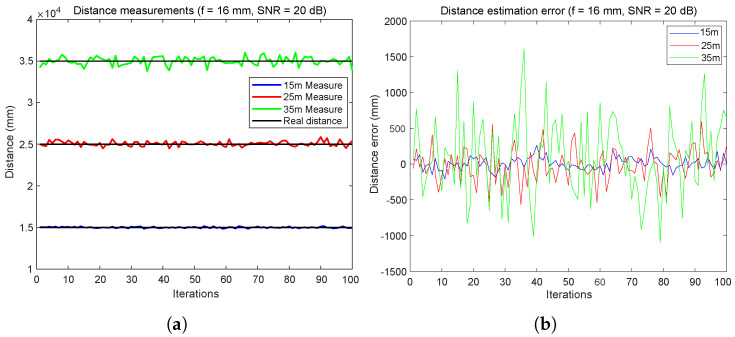
Inter-vehicular distance error estimation using FDMA: error with 20 dB of SNR at focal = 16 mm. (**a**) Distance measurements. (**b**) Distance error estimation.

Figure 9 illustrates the accuracy and reliability of a distance measurement system across different distances.

In Figure 9a, the system’s performance is evaluated over 100 iterations at 15 m, 25 m, and 35 m. The results show high accuracy and minimal variance at the shorter distances of 15 m and 25 m, suggesting the system’s effectiveness for critical applications like vehicle localization and collision avoidance. At 35 m, although the variance increases, the system maintains a practical error margin, demonstrating its potential for longer-distance applications within certain limits.

In Figure 9b, the variability of measurement errors for the same distances over 100 iterations is depicted. The errors for the 15 m distance remain within a range of ±200 mm, indicating stable and acceptable accuracy for short-range vehicle-to-vehicle (V2V) communications. At 25 m, the error expands to approximately ±400 mm, and at 35 m, the error amplitude occasionally exceeds ±1500 mm. These findings reflect the expected increase in measurement challenges with distance but also highlight the system’s overall capability to provide useful distance estimates within real-world operational ranges.

**Figure 10:**
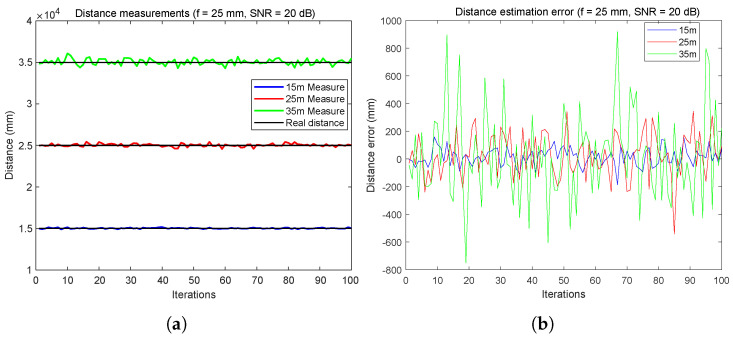
Inter-vehicular distance error estimation using FDMA: error with 20 dB of SNR at focal = 25 mm. (**a**) Distance measurements. (**b**) Distance error estimation.

According to Figure 10, by changing the focal length to 25 mm, we see a better result with a distance very close to the real distance and the oscillations are almost null, especially for distance = 35 m (Figure 10a).

**Figure 11:**
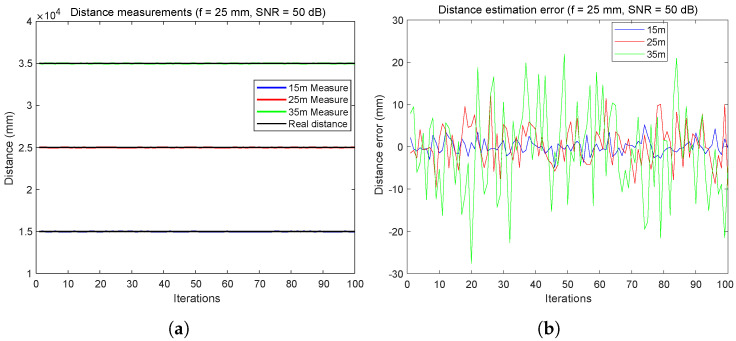
Inter-vehicular distance error estimation using FDMA: with SNR = 50 dB and f3 = 25 mm (**a**) Distance measurements. (**b**) Distance error estimation.

From Figure 11, using a focal length of f3 = 25 mm and a high SNR = 50 dB, it can be clearly seen that the system offers satisfactory accuracy over the entire distance range up to 35 m. The lack of major bias in any of the three target distances in Figure 11a suggests good reliability of the measurement system over this range of distances. Figure 11b shows a slight increase in error with distance, but this remains within acceptable limits for applications requiring millimeter-level accuracy.

**Figure 12:**
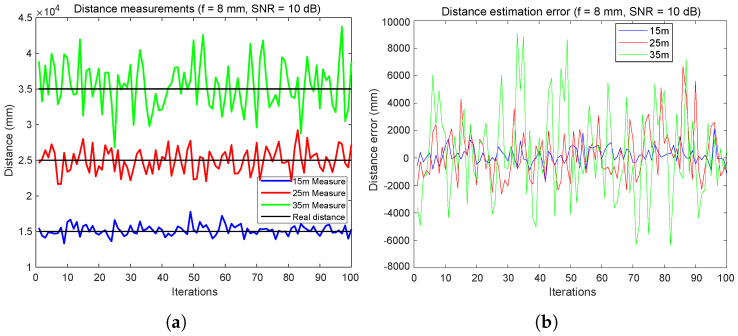
Inter-vehicular distance error estimation using FDMA: with SNR = 10 dB and f = 8 mm. (**a**) Distance measurements. (**b**) Distance error estimation.

Figure 12 depicts an extremely unfavorable scenario characterized by the use of 8 mm focal length and an SNR of 10 dB. It can be observed that the errors increase significantly, reaching values of 4 m for a distance of 25 m.

From the results shown, it can be concluded that increasing the focal length can significantly reduce distance measurement errors, yielding relatively low errors even for a very poor SNR, as indicated in Section 5.1. With an SNR of 50 dB, it is observed that the distance measurement errors are below 3 cm for a distance of 35 m. However, once the data from Figure 12 were analyzed, it can be concluded that under such unfavorable SNR conditions as 10 dB and using such a small focal length, the system does not operate as precisely. Errors of up to 4 m for 25 m and distance measurement errors exceeding 10 m for 35 m were observed in some cases, which may not be suitable for many applications. Therefore, it is advisable to choose the focal length carefully based on the real conditions of each system and application.

#### 5.2.3. Influence of Focal Length and SNR on the Error in Distance Estimation

**Figure 13:**
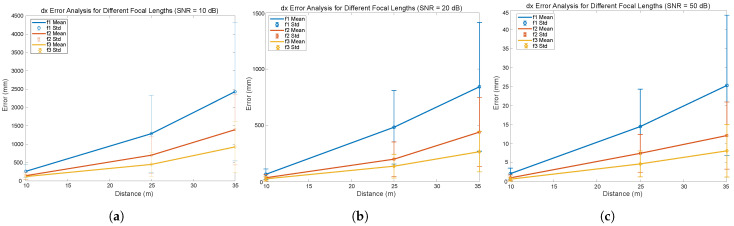
Distance mean error for different focal length f1 = 8 mm, f2 = 16 mm, f3 = 25 mm with (**a**) SNR = 10 dB, (**b**) SNR = 20 dB, and (**c**) SNR = 50 dB.

**Figure 14:**
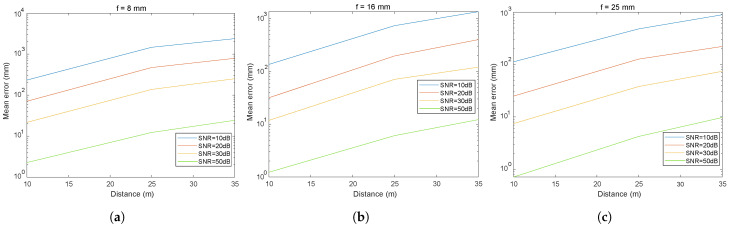
Mean error in the inter-vehicular distance for different focal length with (**a**) f1 = 8 mm, (**b**) f2 = 16 mm, and (**c**) f3 = 25 mm.

**Figure 15:**
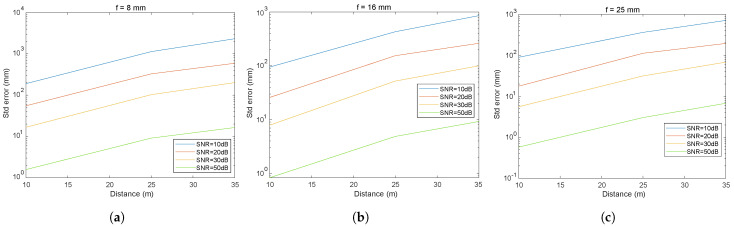
Standard deviation of the inter-vehicular distance error for different focal length with (**a**) f1 = 8 mm, (**b**) f2 = 16 mm, and (**c**) f3 = 25 mm.

To analyze in more detail the influence of the choice of focal length and SNR, a series of results will be presented below. Figure 13, Figure 14 and Figure 15 illustrate the impact of the focal length and SNR on the error metrics, including the average error and the variability (standard deviation), in inter-vehicular distance estimation. In Figure 13, the variation in the distance measurement error between vehicles is depicted, with a specific SNR fixed while varying the focal length and the distance between vehicles. Figure 14 and Figure 15 illustrate both the mean error and the standard deviation of the distance measurement error between vehicles, with the focal length fixed while varying the SNR and the distance between vehicles. We have chosen to present these three figures as they allow for a graphical analysis of the influence of the different parameters.

By analyzing the curves shown, we can observe the effect of three different focal lengths (f1, f2, and f3 corresponding to 8, 16, and 25 mm, respectively) and SNR on the error measured in millimeters as a function of the distance in meters.

Analysis of the results presented in Figure 13, Figure 14 and Figure 15 reveals that under conditions with a high SNR (SNR = 50 dB), errors for all three focal lengths remain below 3 cm even at a distance of 35 m. Furthermore, an increase in the focal length is seen to reduce errors proportionately. In noisy environments (SNR = 20 dB), an escalation in error is evident, with the standard deviations of the distance errors reaching approximately 60 cm for an 8 mm focal length and a distance of 35 m, while hovering around 25 cm using a 25 mm focal length. Under conditions of high noise (SNR = 10 dB) at a distance of 35 m and employing an 8 mm focal length, the standard deviation of the distance error is approximately 2 m, decreasing to 70 cm for a 25 mm focal length.

Upon reviewing the results, it can be concluded that selecting the appropriate focal length is crucial. In low-noise environments, errors are minimal, yet even in highly noisy environments (10 dB), errors in distance measurement could be deemed acceptable for numerous applications. This outcome underlines the beneficial effect of increased focal length on V2V-VLC distance measurement accuracy, which is beneficial for V2V systems where precise directional communication is essential. These results are essential for the development of accurate and reliable V2V communication systems, indicating that adjustments in optical design can be crucial for optimizing the performance of VLC systems in automotive applications.

These results are extremely promising, as they suggest that VLC can achieve centimeter accuracy under high SNR conditions, well beyond what is often required for secure and reliable V2V applications [29].

#### 5.2.4. Impact of Distance between Transmitters on Inter-Vehicular Distance Estimation

One of the factors that may influence the accuracy of the distance measurement between vehicles is the distance between the taillights (or headlights) of the emitting vehicle. Initially, the distance between the taillights of the vehicles cannot be known (assuming that the two cars have not exchanged information with VLC). Therefore, what has been analyzed is how this uncertainty affects the measurement error of the distance between vehicles. For this purpose, the simulations considered a fixed distance between taillights, based on the average distance between the taillights of vehicles. Considering this fixed distance, tests were conducted where the actual distance between the taillights was varied, and the distance between the vehicles was calculated.

**Figure 16:**
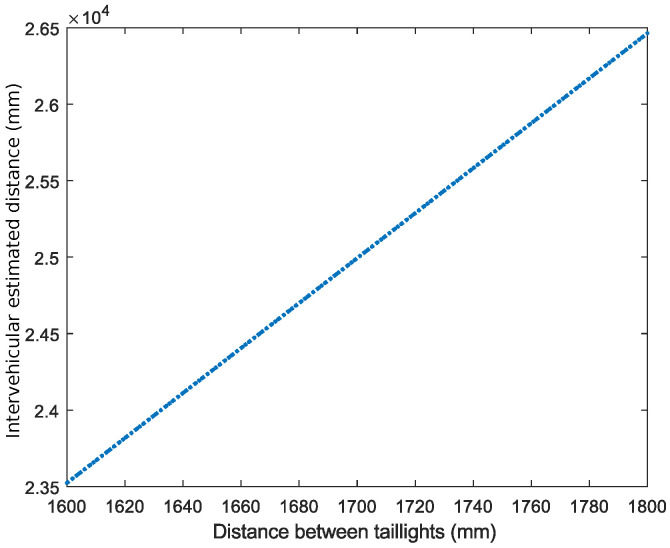
The impact of distance between transmitters on inter-vehicular distance estimation (SNR = 50 dB).

Figure 16 highlights a direct linear correlation between the measurement error in the distance between a vehicle’s taillights and the estimated error in the inter-vehicular distance. The results show that, although the distance between the emitters varies from 1600 mm to 1800 mm, the error associated with estimating the distance between vehicles remains notably low, of the order of a few millimeters.

#### 5.2.5. Lateral Distance Estimation

**Figure 17:**
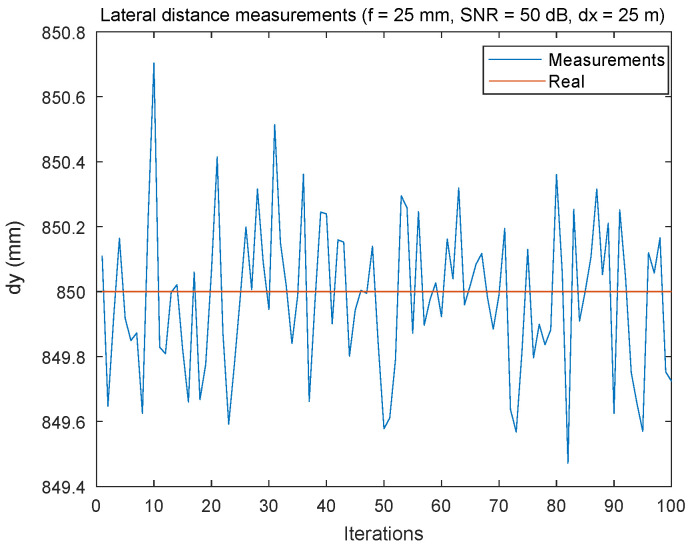
Lateral distance estimation error using FDMA: with SNR = 50 dB, f3 = 25 mm, and dx = 25 m.

Figure 17 shows the results of a simulation evaluating the accuracy of lateral distance measurements at an inter-vehicular distance (dx = 25 m), using a focal length of 25 mm and a high SNR of 50 dB. The data show that, although the actual value of the lateral distance is constantly fixed at 850 mm (represented by the horizontal red line), the simulated measurements (illustrated by the blue line) fluctuate slightly around this fixed value, with deviations of the order of ±0.7 mm.

**Figure 18:**
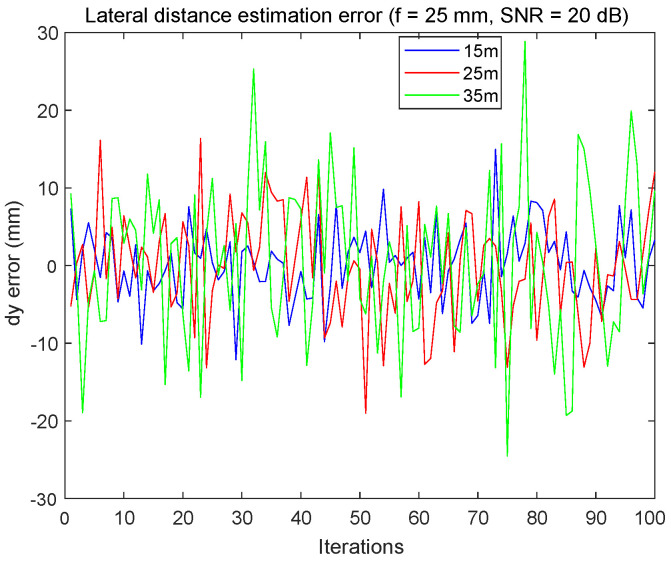
Lateral distance estimation error using FDMA, with SNR = 20 dB and f3 = 25 mm.

Figure 18 effectively shows that, even in an unfavorable SNR = 20 dB environment, the maximum error in the lateral distance estimates remains below 30 mm for all distances tested.

**Figure 19:**
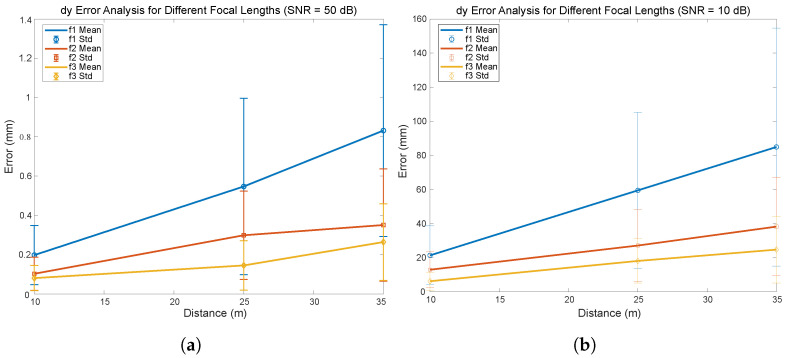
Lateral distance error for different focal length f1 = 8 mm, f2 = 16 mm, f3 = 25 mm, (**a**) SNR = 50 dB and (**b**) SNR = 10 dB.

Figure 19 shows the impact of different lens focal lengths of 8 mm (f1), 16 mm (f2), and 25 mm (f3) on the error in lateral distance estimation as the actual distance increases from 10 to 35 m. The results are expressed in terms of the mean error (represented by solid lines) and the standard deviation around this mean (represented by vertical error bars). Figure 19a reveals that the error in lateral distance estimation varies slightly but significantly across the range of measured distances from 10 to 35 m. For f1 (8 mm), although the error increases with distance, it remains below 0.5 mm, indicating a relatively small variation. The f2 (16 mm) shows a similar trend, with the error increasing slightly but not exceeding 1.0 mm. More notably, the f3 (25 mm) shows the best performance, with a very modest increase in error, reaching a maximum of around 1.4 mm at the furthest distance. In Figure 19b, it can be observed that the errors increase analogously to what has been observed in the errors for the inter-vehicle distance measurement. It is observed that for a distance of 35 m, the error is around 10 cm for a focal length of 8 mm, reducing to a value of approximately 2.5 cm for a focal length of 25 mm.

This precision in error, particularly with the 25 mm focal length, bodes well for applications where safety depends greatly on accurate lateral positioning, such as maintaining a safe distance between vehicles, detecting blind spots, or ensuring coordinated maneuvers in autonomous driving scenarios. The low variability of errors, as shown by the small standard deviations, also underlines the reliability of these measurements, a crucial aspect for systems where decisions need to be made in real-time and with great confidence.

### 5.3. Experimental Results

To validate the results obtained from the simulations, several experimental tests were conducted. The setup was as follows: 100 measurement repetitions of 0.04 s each were acquired, similar to what was performed in the simulations.

**Figure 20:**
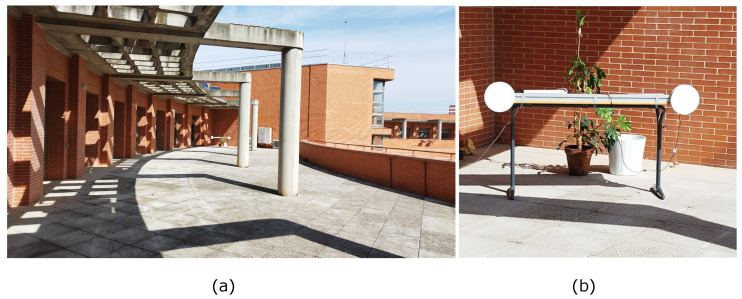
Emitter configuration used in the experimental test. (**a**) Environment setup and (**b**) emitters setup.

Two emitters of 5 W power each were used and positioned at a distance of 1.5 m between them. The emitters were positioned 0.8 m above the ground. The emission frequencies of each emitter were 6 kHz and 9 kHz, limited by the driver used for this test. The emitters are shown in Figure 20.

**Figure 21:**
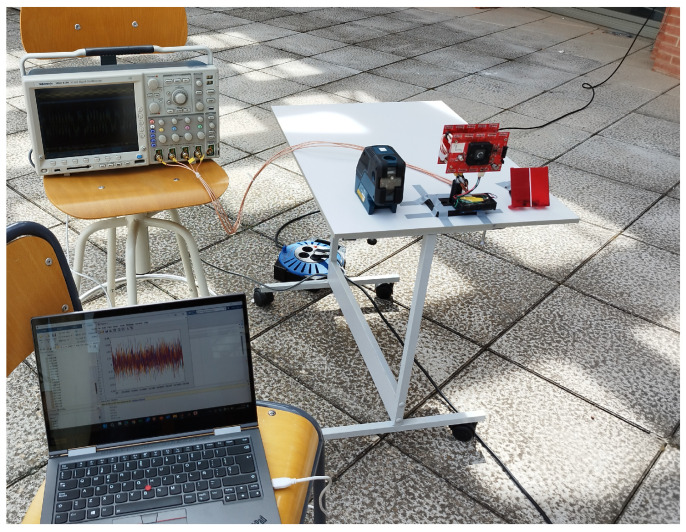
Setup of the receiver with the oscilloscope used in the experimental test.

For the experimental tests, board 2 described in the previous sections was used, consisting of a Hamamatsu S5991-01 PSD coupled with a lens with a focal distance of 6.3 mm. The output signal of the PSD was amplified with a gain of 2 million and captured with an oscilloscope Tektronix MSO4104 connected to a PC with a sampling frequency of 250 kHz. The setup of the receiver with the oscilloscope is depicted in Figure 21. The distance between the tail-light emitters and the receiver was 25 m. For an accurate distance measurement setup of 25 m, a Leica DISTO D2 laser rangefinder with a resolution error of ±1.5 mm and a Boch GCL 25 were used.

In addition to multiple simulations carried out to show the behavior of the system, a simulation reproducing the same conditions as the experimental setup was performed.

**Figure 22:**
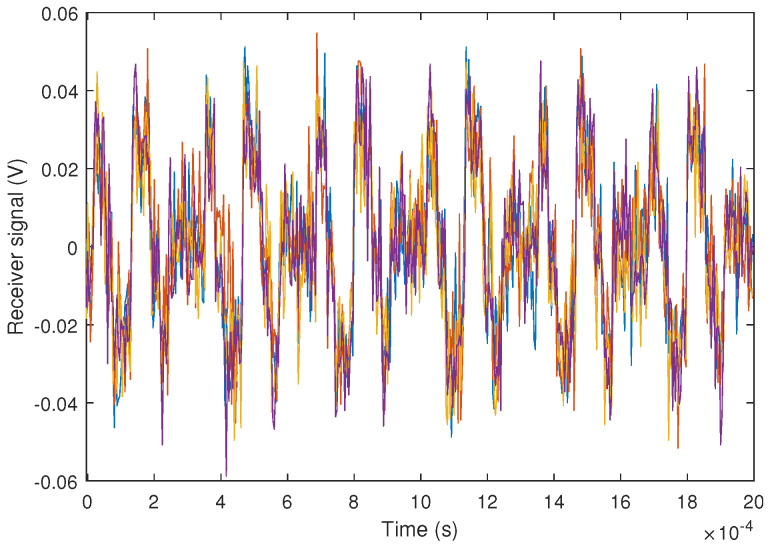
Results of the experimental test at 25 m and SNR = 10 dB. Experimental signal received by each of the PSD channels (colors shown in the figure correspond, each one, to the signal obtained from each channels of the PSD).

Figure 22 shows the signal received by each of the PSD channels, once amplified, at a distance of 25 m between the emitters and the receiver. The SNR of the received signal is approximately 10 dB.

**Figure 23:**
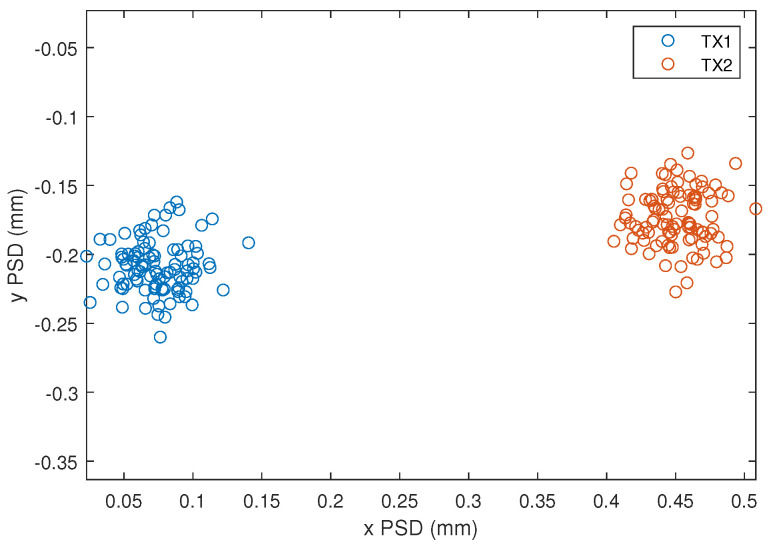
Results of the experimental test at 25 m and SNR = 10 dB. Impact points on the surface of the PSD from the two emitters.

From the processing of the PSD signals, the impact points on the surface of the PSD receiver from the two emitters are obtained, as shown in Figure 23.

**Figure 24:**
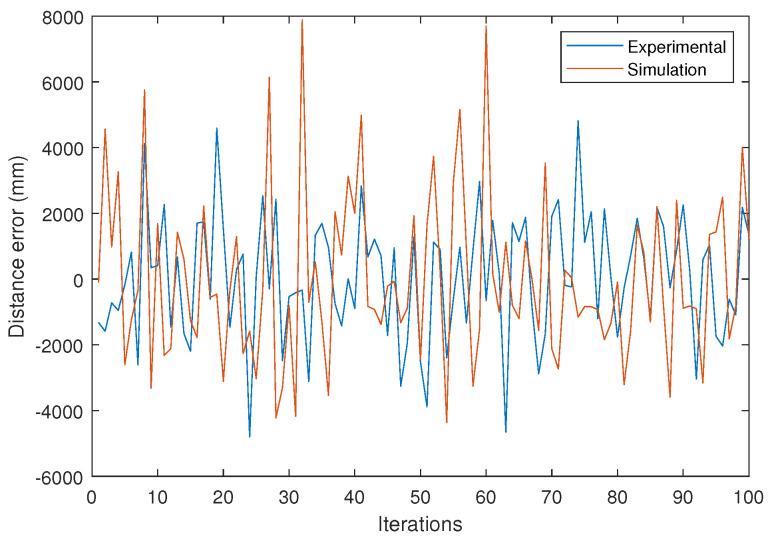
Results of the experimental test at 25 m and SNR = 10 dB. Error in distance measurement on experimental tests (blue) and simulation results (orange).

From the impact points, and according to Equation (10), the distance measurement results are obtained. Figure 24 shows the error in distance measurement for the 100 measurements. Additionally, the result obtained from our simulator is shown in orange lines.

In addition to the test conducted at 25 m, another experimental test was performed under the same conditions but with a variation in the distance between vehicles to 10 m. At this distance, an SNR of 18 dB was measured. The results will be presented similarly to the previous case.

**Figure 25:**
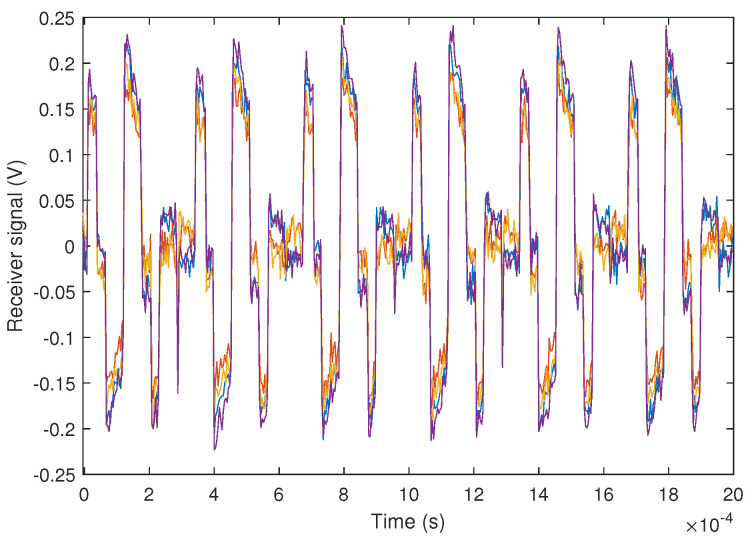
Results of the experimental test at 10 m and SNR = 18 dB. Experimental signal received by each of the PSD channels (colors shown in the figure correspond, each one, to the signal obtained from each channels of the PSD).

Firstly, in Figure 25, the received signal for each channel of the PSD is depicted. As can be observed, the signal is much more defined than in Figure 22 due to the improved SNR.

**Figure 26:**
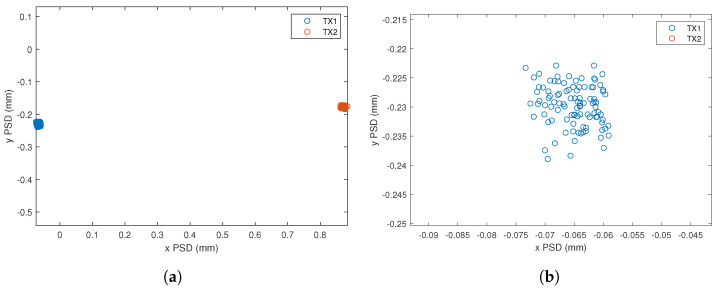
Results of the experimental test at 10 m and SNR = 18 dB. (**a**) Impact points on the surface of the PSD from the two emitters. (**b**) Zoom of the impact point of the emitter 1.

Figure 26 shows the impact points of the two emitters on the surface of the PSD. Since the distance between the emitters and the receiver is shorter, from 25 m to 10 m, the separation of the images of each emitter on the surface of the PSD is greater than the results shown in Figure 23. Please note the values on the scales.

**Figure 27:**
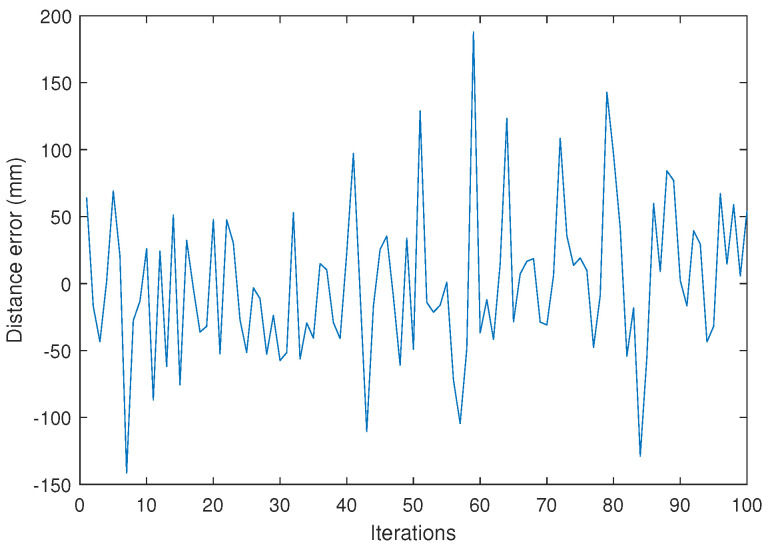
Results of the experimental test at 10 m and SNR = 18 dB. Error in distance measurement on experimental tests.

Finally, Figure 27 displays the results of the error in the distance measurements between vehicles obtained from the experimental signals.

To analyze the obtained results, attention will be focused on the standard deviation values of the error in the vehicle distance measurement, as the mean error could be compensated with proper calibration. In the experimental results, with a distance between vehicles of 25 m, a standard deviation in vehicle distance measurement of 1.875 m is obtained. Under the same conditions in the simulations, a standard deviation of 2.524 m is obtained. Both measures are very similar and are in the range of values shown in previous simulations, as can be seen, for example, in Figure 15a. The differences between the results shown in this figure, conducted with an 8 mm focal length, 25 m distance between vehicles, 10 dB SNR, and a distance of 1.7 m between emitters, and those obtained in this section are mainly due to reducing the focal length from 8 to 6.3 mm and reducing the distance between the emitters from 1.7 m to 1.5 m. From Figure 15a, the obtained standard deviation value of the inter-vehicle distance measurement error is 1.45 m. Since the focal distance is larger (error decreases as the focal distance increases) and the emitter separation distance is greater (greater emitter separation leads to less triangulation error), the obtained error is lower than that obtained in the experimental tests. If we focus on the experimental results from Figure 27 obtained with a distance between vehicles of 10 m, an SNR of 18 dB was measured. The standard deviation of the inter-vehicle distance error was calculated to be 56.7 mm. This value can be compared with the simulation results shown in Figure 15a at a distance of 10 m, focal length of 8 mm, and an SNR of 20 dB, where a standard deviation of the distance error of 54 mm is obtained. As in the previous case, the small differences observed are due to the fact that the conditions are not exactly the same.

Nevertheless, even in such an unfavorable environment as shown, distance measurement errors of the order of 2 m are achieved at a distance of 25 m and errors below 6 cm for distances of 10 m, considering that a processing time of 0.04 s is used. In other words, 10,000 signal samples acquired at 250 kHz are processed, allowing for 25 distance measurements between vehicles per second. This could be acceptable for many applications. It is worth noting that if it were necessary to reduce this distance error, more signal processing time could be used. For example, if 10 times more processing time were used, i.e., 0.4 s (obtaining a distance measurement rate per second of 2.5), the typical deviation of the real measurements at 25 m would be 679.6 mm.

Based on the results obtained in the experimental test and comparing them with the simulation under the same conditions and with the rest of the simulations, taking into account the parameters used in each of them, it can be concluded that the simulations conducted are valid, and the results shown would be fairly close to the real ones.

## 6. Conclusions

This paper proposed a new positioning V2V-VLP system for an outdoor environment using AoA measurement and based on a single PSD sensor. We have evaluated the effect of the focal length and SNR variation on the distance estimation performance.

Given the results presented, it can be concluded that increasing the focal length can significantly reduce the inter-vehicle distance measurement errors, resulting in relatively low errors even at poor SNR. Similarly, for lateral distance estimation, the error remains below 30 mm for all distances tested with a focal length of 25 mm in low-noise environments. These findings, also demonstrate that the accuracy of our system for estimating the distance between vehicles is robust and independent of variations in the distance between taillights (emitters), thus affirming its potential for reliable use in automotive safety systems.

We have validated the simulation results with experimental tests conducted under realistic conditions. The obtained results confirm that the simulations accurately reproduce reality.

The results presented demonstrate that sub-centimeter accuracies can be achieved under conditions of good SNR. Under unfavorable conditions, with very low SNR and a very small focal length, errors in vehicle distance measurement of the order of 2 m for a distance of 25 m were obtained in both simulations and experimental tests. Another experimental test conducted at a distance of 10 m between vehicles, under SNR conditions of 18 dB, yielded a standard deviation of the inter-vehicle distance measurement error of less than 6 cm. This occurred with a distance measurement rate of 25 measurements per second, and the error could be reduced by lowering the rate of distance measurements per second. These results make the system viable for a wide range of V2V applications.

The purpose of this approach is to confirm the reliability of our system and validate the relevance of VLC communication as a future solution for precise positioning in vehicular communications.

## Figures and Tables

**Table 1:** Simulation parameters.

Parameter	Value
SNR	20 dB, 50 dB
Focal length	8 mm, 16 mm, 25 mm
Modulation	FDMA
Frequency rate	1 MHz
Iteration number	100
Communication distance	15–35 m
Pt	15 W
w	1.7 ± 0.1 m
Receiver active area	$5.0671\times 10^{-4}$ m^2^
Processing time	0.04 s

## Data Availability

The data supporting the conclusions of this article will be made available by the authors on request.

## Abbreviations

The following abbreviations are used in this manuscript:

AOA	Angle of Arrival
LOS	Line of Sight
FoV	Field of View
FDMA	Frequency Division Multiple Access
SNR	Signal-to-Noise Ratio
std	Standard Deviation
LED	Light Emitting Diode
PSD	Position Sensitive Device
VLC	Visible Light Communication
VVLP	Vehicular Visible Light positioning
V2V	Vehicle-to-Vehicle
